# Effective Denoising of Multi-Source Partial Discharge Signals via an Improved Power Spectrum Segmentation Method Based on Normalized Spectral Kurtosis

**DOI:** 10.3390/s25123798

**Published:** 2025-06-18

**Authors:** Baojia Chen, Kaiwen Li, Yipeng Guo

**Affiliations:** 1 Hubei Key Laboratory of Hydroelectric Machinery Design and Maintenance, China Three Gorges University, Yichang 443002, China; gypeng1999@163.com; 2 College of Electrical Engineering & New Energy, China Three Gorges University, Yichang 443002, China; likaiwen066@126.com

**Keywords:** transformer, multi-source partial discharge, improved power spectrum segmentation, power spectral kurtosis

## Abstract

In the field of partial discharge (PD) analysis, traditional methods typically employ single-source PD signal-processing techniques. However, these approaches exhibit significant limitations when applied to transformers with relatively complex structures. To overcome these limitations and achieve precise characterization of composite PD signatures, this study proposes an improved power spectrum segmentation method (IPSK) based on spectral kurtosis. Firstly, normalized power spectral kurtosis is used to select the appropriate parameters. Then, through the improved power spectrum segmentation method, the segmentation frequency band with the least noise is obtained. Finally, the instantaneous signal components with physical significance are obtained by reconstructing each frequency band through inverse fast Fourier transform. By analyzing the simulated signals and measured data of partial discharge, the proposed method is compared with EWT, AEFD, VMD, and CEEMDAN. The results show that IPSK has a good suppression effect on noise interference.

## 1. Introduction

The structure and operating conditions of power transformers are complex. During their manufacturing, transportation, installation, commissioning, and operation, typical insulation defects such as bubbles, burrs, air gaps, surface contamination, and poor contact are prone to occur [1]. These defects can lead to localized electric field breakdown on the surface or inside the transformer, resulting in partial discharge (PD) phenomena [2]. PD signal detection can enable early warning and accurate diagnosis of insulation faults in transformers [3]. However, in practical engineering, the detected PD signals are often contaminated with significant noise interference, which severely affects the effectiveness and accuracy of diagnosis.

To effectively suppress background noise in PD signals, extensive research has been conducted by scholars both domestically and internationally in recent years. Huang et al. [4] proposed empirical mode decomposition (EMD), which offers a novel adaptive decomposition technique for processing PD signals. However, this method is plagued by severe mode mixing and substantial noise interference. To address these issues, Jin et al. [5] introduced a PD denoising method for switchgear based on adaptive EEMD, which significantly outperforms traditional wavelet algorithms and EMD methods. Nevertheless, EEMD still exhibits residual noise interference and high computational complexity.

To further improve the mode mixing and spurious components in the decomposition process, scholars have proposed complete ensemble empirical mode decomposition with adaptive noise (CEEMDAN). Sun et al. [6] employed the CEEMDAN with adaptive noise method, which effectively removed residual white noise interference but failed to fundamentally resolve the mode-mixing problem. For improved mode mixing, the variational mode decomposition (VMD) method proposed by Zosso et al. [7] achieved effective analysis of complex noise signals by leveraging the characteristics of finite bandwidth and unique central frequency. Building on this, Xia et al. [8] introduced an improved VMD method for PD signal denoising, yet practical applications still face challenges in parameter selection. Xiao et al. [9] proposed a method to determine the optimal number of modes *N* based on the number of peaks in the signal spectrum, which is primarily aimed at analyzing low-frequency oscillations in power systems and does not fully account for the impact of noise on signals. Therefore, it has limitations in PD signal processing.

Other methods include Gilles et al.’s [10] proposed empirical wavelet transform (EWT) method, which presets boundary frequencies by analyzing the distribution of peaks in the signal spectrum and constructs orthogonal filter banks to decompose signals by segmenting the spectrum into different bands. Ma et al. [11] combined the improved empirical wavelet transform (IEWT) with the Fourier spectrum segmentation method, which essentially solved the mode-mixing problem during PD signal decomposition but exhibited drawbacks such as low efficiency in selecting characteristic components and high computational load. Zheng et al.’s [12] adaptive empirical Fourier decomposition (AEFD) successfully decomposes non-stationary signals, yet requires manual spectrum boundary setting, introducing practical implementation uncertainties.

To address these problems, an improved power spectrum segmentation (IPSK) method based on spectral kurtosis is proposed in this paper. By introducing the improved power spectrum segmentation technology, the narrowband interference and white noise components in multi-source complex PD signals can be effectively filtered out, which improves the problem of insufficient decomposition accuracy of traditional methods in processing multi-source composite PD signals. To verify the effectiveness of the method, this paper selects typical methods such as EWT, AEFD, VMD, and CEEMDAN for comparative analysis, and it proves the superiority of the IPSK method in extracting PD signals.

## 2. Basic Theory

### 2.1. Common Spectrum Segmentation Methods

Precision in spectral segmentation is crucial for the success of decomposition techniques like EWT and AEFD. Common spectrum segmentation methods include local maxima, local minima, and ε neighborhood methods. EWT employs the local maxima method, which determines segmentation boundaries by locating local maxima points and their midpoints in the spectrum. While straightforward, this method may suffer from a reduced segmentation accuracy in complex spectrum structures, especially when multiple maxima are close to each other. Conversely, AEFD tends to use the ε neighborhood method, which adaptively searches for boundaries based on an initial boundary set. However, the ε neighborhood method exhibits a certain degree of subjectivity in determining the ε value. Different ε values can lead to distinct segmentation results, thereby increasing the uncertainty and adjustment the complexity of the method. Given that transformer PD signals are highly susceptible to noise, the spectrum segmentation methods of EWT and AEFD have limitations in practical applications, making precise segmentation challenging.

To illustrate the differences in spectrum segmentation effects between EWT and AEFD, a simulated signal is used for comparative analysis. The simulated signal consists of two amplitude-modulated signals, a sinusoidal signal, and Gaussian white noise n(t) with a signal-to-noise ratio (SNR) of −5 dB, expressed as follows:

(1) $$\begin{array}{l} x(t)=x_{1}(t)+x_{2}(t)+x_{3}(t)+n(t)\\ \;\;=\sin(16\times 10^{6}\pi t)+(\cos(1.5\times 10^{6}\pi t))\;\sin(32\times 10^{6}\pi t)\\ \;\;\;\;\;+(1.2+\cos(2\times 10^{6}\pi t))\sin(60\times 10^{6}\pi t)+n(t) \end{array}$$

**Figure 1:**
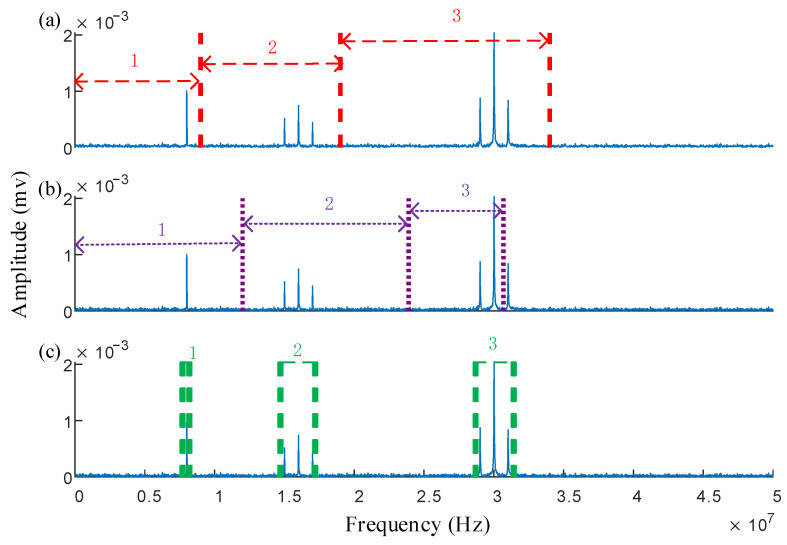
Comparison of spectrum segmentation results of simulated signals: (**a**) AEFD, (**b**) EWT, (**c**) IPSK.

The segmentation results of EWT and AEFD are shown in Figure 1a,b, respectively. As can be seen in the figures, EWT incorrectly splits the signal into two parts, with clearly unreasonable segmentation boundaries. AEFD, on the other hand, results in overly broad segmentation intervals, leading to significant noise interference within each frequency band, which directly affects the accuracy of the reconstructed signal after decomposition and filtering. Since real transformer PD signals also contain substantial noise, optimizing spectrum segmentation boundaries and reducing noise interference within each frequency band are critical for accurate signal feature extraction.

Unlike EWT and AEFD, IPSK does not perform continuous segmentation across the entire frequency range but instead acts as a set of discrete filter banks. The center frequencies of the filters correspond to the first few maximum amplitude frequencies, and the upper and lower cutoff frequencies of each filter are determined by the amplitude relationships between the center frequency and its adjacent frequencies. As shown in Figure 1c, IPSK achieves more reasonable frequency band division, effectively extracting the main signal features while minimizing noise interference.

### 2.2. Improved Welch Power Spectrum Segmentation Methods

**Figure 2:**
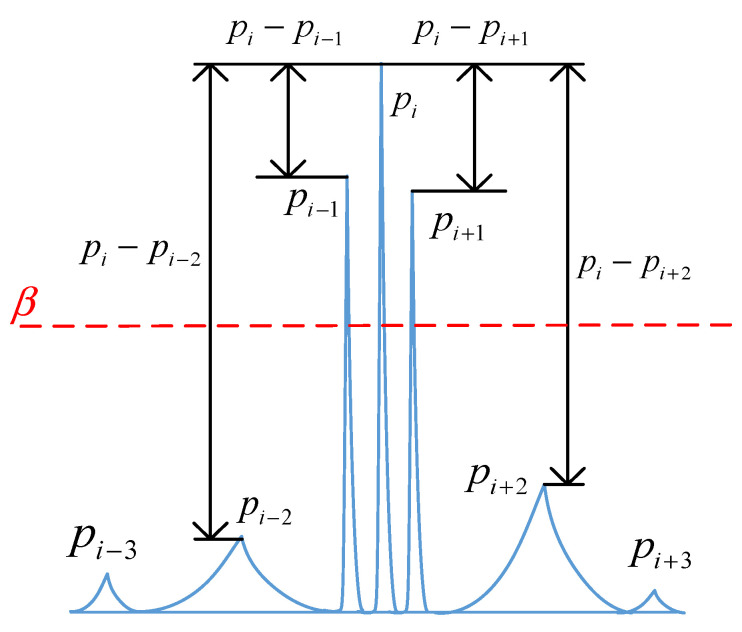
Relationship between center frequency amplitude and side frequency variation.

In normal measurements, electrical signals are often subject to noise interference, especially PD signals, which are primarily affected by narrowband interference and white noise. While the traditional Welch method is widely adopted due to its simplicity and computational efficiency, its conventional segmentation approach exhibits limitations in identifying nonlinear signal characteristics, suppressing noise, and effectively separating PD signals [13]. To overcome these limitations and enhance signal-processing performance, an improved Welch power spectrum segmentation method is proposed. By incorporating normalized spectral kurtosis, this approach significantly improves the detection of nonlinear signal features, enhances noise suppression and signal separation, and optimizes the accuracy and stability of power spectrum segmentation, making it more suitable for processing complex electrical signals such as PD signals. The specific steps are as follows:

(1) Calculate the signal segmented power spectrum: Divide the signal $x\left(n\right)$ into *L* segments. If each segment overlaps by half, then $L=\left(N-M/2\right)/\left(M/2\right)$, where *N* is the length of each segment and *M* is the number of overlapping points. The power spectrum $P_{PER}^{i}(\omega )$ of each segment is calculated as
  (2) $$P_{PER}^{i}(\omega )=\frac{1}{U}{\left|\sum_{n=0}^{N-1}x_{N}^{i}(n)d_{2}(n)e^{-jwn}\right|}^{2}$$
  (3) $$U=\frac{1}{N}\sum_{n=0}^{N-1}d_{2}^{2}(n)$$
  where U is the normalization factor, $d_{2}(n)$ is the window function, and $x_{N}^{i}(n)$ is the *i*-th segment of the signal.
(2) Calculate the average power spectrum $P_{PER}(\omega )$:
  (4) $$P_{PER}(\omega )=\frac{1}{L}\sum_{i=1}^{L}{\hat{P}}_{PER}^{i}(\omega )=\frac{1}{UL}\sum_{i=1}^{L}{\left|\sum_{n=0}^{N-1}x_{N}^{i}(n)d_{2}(n)e^{-jwn}\right|}^{2}$$
(3) Determine the center frequencies and boundary positions of each frequency band: Extract all local maxima $p_{i}$ and their positions $f_{i}$ from $P_{PER}(\omega )$, and compile this information into a set $P=\left\{(f_{i},p_{i}),\;i=1,2,\cdots J\right\}$, where *J* represents the total number of maxima in the power spectrum. Based on the distribution of the power spectrum, select the locations of the top *K* maxima as the center frequencies of the frequency bands. Then, determine the upper and lower sideband frequencies according to the amplitude variation relationships. The steps are as follows:
  (1) Determine the amplitude threshold β for boundary selection:
    (5) $$\beta =\left(1-w\right)p_{i}\;\;\;\;\;\;\;\;\;w\subset \left[0,1\right]$$
    where *w* is the bandwidth control coefficient, determined as described in Section 2.3.
  (2) Determine the center frequency and boundary positions. Taking $p_{i}$ as the center, $p_{i}$ is compared with its adjacent extreme value $p_{i+1}$ on the right side. If $\left|p_{i}-p_{i+1}\right|\geq \beta$, the corresponding frequency of $p_{i+1}$ is taken as the upper side frequency position. If the above conditions are not met, the next adjacent extreme value on the right side of $p_{i}$ is compared with the $p_{i}$ amplitude to determine whether it is greater than the threshold β. Repeat this process until the condition is met. The lower boundary is determined similarly. The relationship between the center frequency amplitude and boundary amplitude is illustrated in Figure 2.

### 2.3. Improved Welch Power Spectrum Segmentation Methods’ Selection Based on Normalized Power Spectrum Kurtosis

Power spectral kurtosis (PSK) is a statistic used to describe the distribution characteristics of the signal power spectrum, which can measure the impact component of the signal power spectrum in the frequency domain. When transient or impact components are present, PSK increases. Since PD signals are primarily impact signals, PSK can be used to extract their features [14]. The proposed threshold selection methodology comprises the following steps:

(1) Power spectral kurtosis calculation: The PSK for segmented signals is computed as
  (6) $$\mathrm{PSK}=\frac{\sum_{n=1}^{N}{\left(F_{i}-F_{mean}\right)}^{4}}{{\left(\sum_{n=1}^{N}{\left(F_{i}-F_{mean}\right)}^{2}\right)}^{4}}$$
  where $F_{i}$ is the power spectrum value at the *i*-th frequency point, and $F_{mean}$ is the average power spectrum value.
(2) Threshold selection based on normalized PSK (NPSK): The optimal segmentation threshold *w* ∈ [0, 1] is determined through the following:
  (1) Grid Search: The interval [0, 1] is discretized with Δ*w* = 0.1 resolution, computing segmented frequency bands for each *w* value.
  (2) NPSK Calculation: For each segmentation result, the average PSK value across all frequency bands is calculated and normalized:
    (7) $$\mathrm{NPSK}(w_{k})=\frac{{\mathrm{PSK}}_{mean}\;(w_{k})-min({\mathrm{PSK}}_{mean})}{max({\mathrm{PSK}}_{mean})-min({\mathrm{PSK}}_{mean})}$$
    Discrete determination criterion for optimal threshold *w*: For ordered discrete points $\left\{(w_{k},NPSK\left(w_{k}\right)\right)$∣k=1,2,⋯,N} forming a polyline, the transition point satisfies
    (8) $$\;\Delta k\;=\;\left|\frac{\Delta y_{k}\;\;}{\Delta x_{k}}-\frac{\Delta y_{k-1}\;}{\Delta x_{k-1}\;}\right|\;\;$$
    where $\Delta y_{k}=\mathrm{NPSK}\left(w_{k}+1\right)-\mathrm{NPSK}\left(w_{k}\right)$ and $\Delta x_{k}=w_{k}+1-w_{k}$.
    The optimal threshold corresponds to the maximum slope variation point:
    (9) $$w=w_{m}$$
    where $m=\arg\max(\Delta_{k}\;)$.
(3) Power spectrum segmentation: The optimal threshold *w* is applied to the segmentation algorithm to obtain the best power spectrum segmentation results.

**Figure 3:**
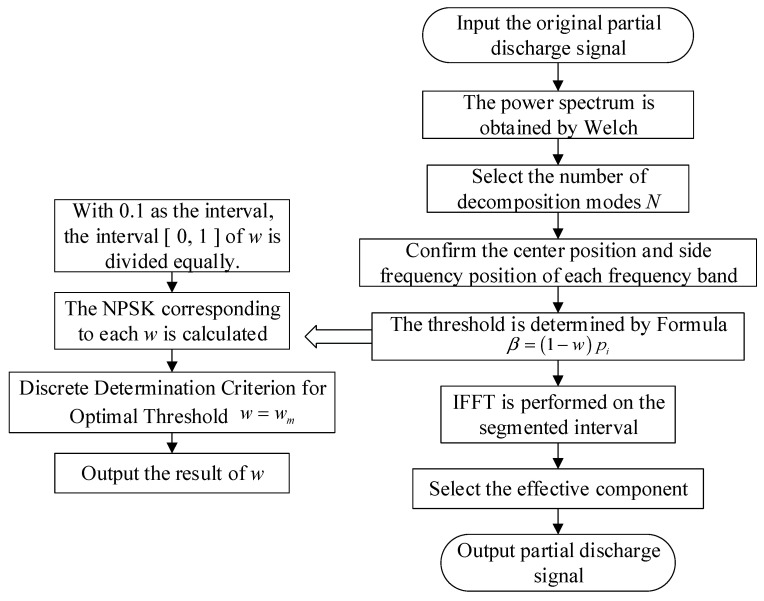
Flow chart of IPSK method.

The flow chart of the IPSK method is shown in Figure 3.

**Figure 4:**
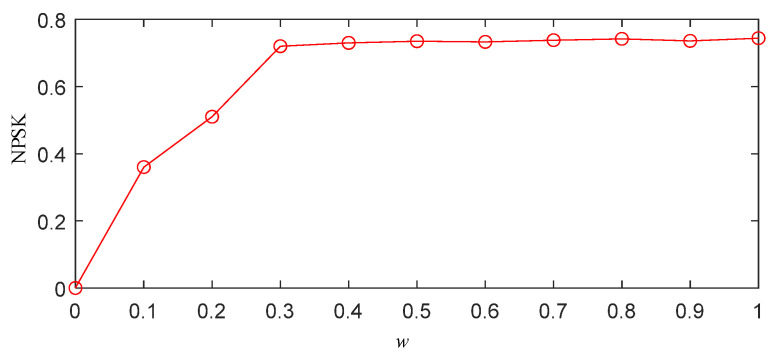
Normalized power spectral kurtosis of simulated signal x(t).

Through the above steps, the optimal threshold *w* of the simulation signal can be obtained, as shown in Figure 4. From Figure 4, it can be observed that as *w* increases, the normalized PSK (NPSK) also rises, indicating the presence of distinct impulsive signals in the segmented frequency bands, which validates the effectiveness of the segmentation. When *w* reaches 0.3, further increases in *w* result in almost no change in the NPSK value. This suggests that the impulsive components in the segmented frequency bands do not increase any further. Continuing to increase *w* would lead to an expansion of the bandwidth of the segmented frequency bands, thereby increasing the white noise content and compromising the segmentation accuracy. Therefore, *w* = 0.3 is identified as the optimal value.

The optimal *w* value is then substituted into β to obtain the best power spectrum segmentation result. To maintain consistency in comparison, the segmentation result of IPSK is converted from the power spectrum to the frequency spectrum, as shown in Figure 1c. Comparative analysis of the results presented in Figure 1 demonstrates that IPSK achieves non-continuous segmentation, enabling precise identification of the target frequency band with minimal noise interference. This performance significantly outperforms both the AEFD and EWT methods in terms of segmentation accuracy and noise suppression capability.

## 3. Simulation Analysis

In order to verify the effectiveness of IPSK, two sets of single exponential oscillation attenuation models and double exponential oscillation attenuation models with different oscillation frequencies are set up to construct multi-source PD signals [15]. To enhance the complexity of the PD signals, two additional sets of attenuation functions were incorporated into one of the models. This signal combines a variety of PD characteristics, including different discharge types and intensities, to simulate the complex discharge conditions that may occur in the actual operation of the transformer. The formula is

(10) $$f_{1}(t)=A_{1}\sin\left(2\pi f_{c1}t\right)\left(e^{-\frac{t_{3}}{\tau_{21}}}+e^{-\frac{1.3t_{4}}{\tau_{22}}}-e^{-\frac{2.2t_{4}}{\tau_{22}}}\right)$$

(11) $$f_{2}(t)=A_{2}\left(e^{-\frac{t_{1}}{\tau_{21}}}+e^{-\frac{1.3t_{2}}{\tau_{22}}}-e^{-\frac{2.2t_{2}}{\tau_{22}}}\right)+A_{2}\sin\left(2\pi f_{c2}t\right)\left(e^{-\frac{t_{3}}{\tau_{21}}}+e^{-\frac{1.3t_{4}}{\tau_{22}}}-e^{-\frac{2.2t_{4}}{\tau_{22}}}\right)$$

In these equations, *A* represents the signal amplitude, τ are the decay coefficients, $f_{c}$ is the oscillation frequency, and $t_{r}\;\;r\in \left[1,2,3,4\right]$ is the time of discharge oscillation, which take values of 5 µs, 13 µs, 20 µs, and 30 µs, respectively.

**Figure 5:**
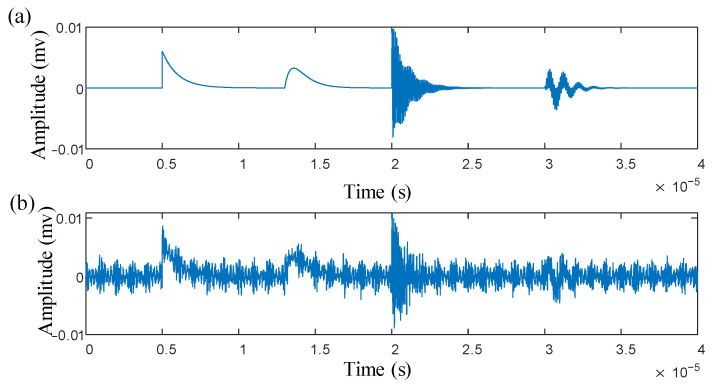
(**a**) Time−domain waveform of multi-source PD signal. (**b**) Time-domain waveform of multi-source PD signal with noise.

In the simulation experiment, the sampling frequency was set to 20 MHz, and the number of sampling points was 4000. The specific parameters of the PD signal are listed in Table 1, and the corresponding time-domain waveform is shown in Figure 5a.

To simulate realistic field measurement conditions, Gaussian white noise with a signal-to-noise ratio (SNR) of −2 dB and narrowband interference were introduced into the generated multi-source complex PD signals. The narrowband interference consists of a combination of sinusoidal signals and amplitude-modulated (AM) signals, mathematically expressed as follows:

(12) $$f_{3}(t)=0.7\sin(20\pi t)+1.2\left(0.8+\cos(2\pi t)\right)\cdot \sin(64\pi t)$$

The specific time-domain waveform of the noisy multi-source PD signal is shown in Figure 5b.

**Figure 6:**
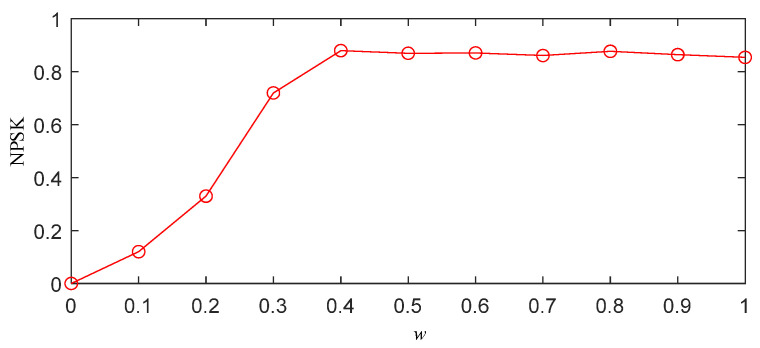
Normalized power spectral kurtosis of noisy PD simulation signal.

To obtain the optimal frequency band segmentation results using the IPSK method for simulated signals, it is necessary to determine the appropriate value of parameter *w*. Following the same procedure described in Section 2.3, we calculated the NPSK as shown in Figure 6. The results demonstrate that the kurtosis reaches its maximum value when *w* = 0.4. Further increasing *w* beyond this value shows negligible changes in the NPSK results. Therefore, the optimal parameter *w* was determined to be 0.4 for subsequent analysis.

**Figure 7:**
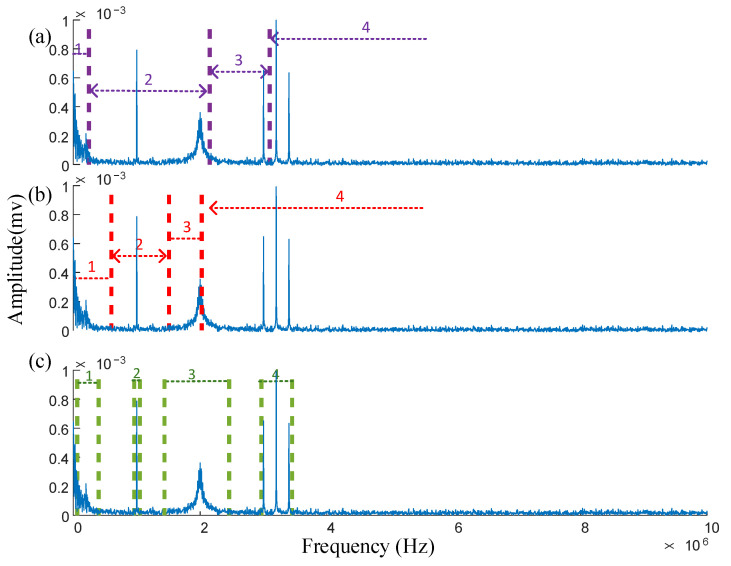
The spectrum segmentation results of the three methods of the simulated signal are: (**a**) EWT, (**b**) AEFD, (**c**) IPSK.

The value of *w* obtained is substituted into the threshold β, yielding the segmentation results for IPSK. These results are compared with the spectral segmentation performance of the EWT and AEFD methods. To ensure consistency in the comparison, the segmentation results of IPSK are transformed from the power spectrum to the frequency spectrum, as illustrated in Figure 7a–c.

As shown in Figure 7a, the EWT failed to segment the amplitude modulation signal, and it incorrectly divided the frequency bands of the two PD signals into two parts. This segmentation error resulted in significant mode mixing during subsequent time-domain analysis, severely compromising the accuracy of signal feature extraction. Figure 7b demonstrates that although the AEFD method successfully separated the sinusoidal and amplitude-modulated signals, it committed segmentation errors when processing the second PD signal by incorrectly assigning its right portion to the amplitude-modulated signal’s frequency band. In contrast, Figure 7c reveals that the IPSK method achieved non-continuous segmentation in the frequency domain, with each segmented band occupying a limited frequency range containing minimal noise. Most importantly, IPSK correctly segmented both PD signals. These comparative results clearly indicate that IPSK provides more rational spectral segmentation than both EWT and AEFD methods, demonstrating a superior performance in processing noisy signals.

**Figure 8:**
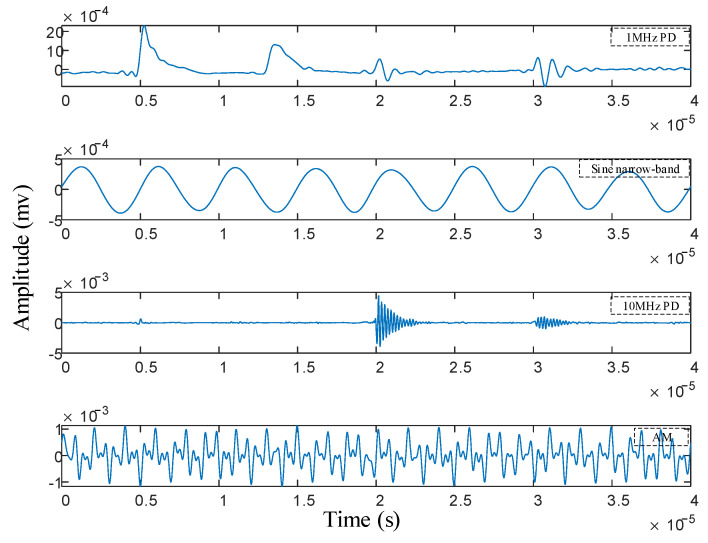
Time-domain waveform reconstruction of IPSK in each frequency band.

In order to feel the spectrum segmentation effect of IPSK more directly, the time-domain waveform reconstruction of each frequency band was carried out, and the results are shown in Figure 8. It can be seen from the diagram that the reconstructed four components correspond to the PD signal with a frequency of 1 MHz, the sinusoidal narrowband interference signal, the PD signal with a frequency of 10 MHz and the amplitude modulation signal. It can be seen from the diagram that each component is clear, and there is no modal mixing phenomenon, indicating that the method accurately decomposes the multi-source complex PD signal into two independent PD signals.

**Figure 9:**
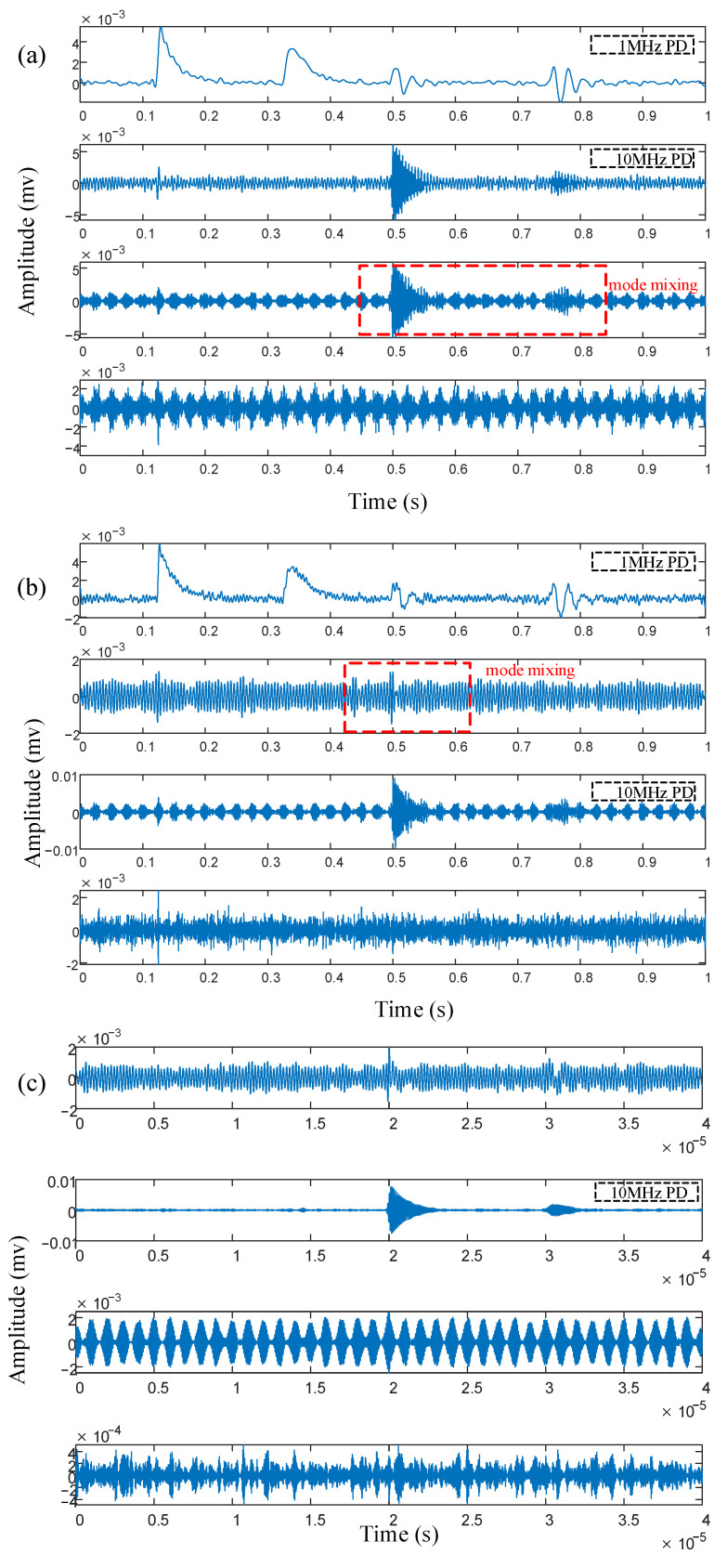

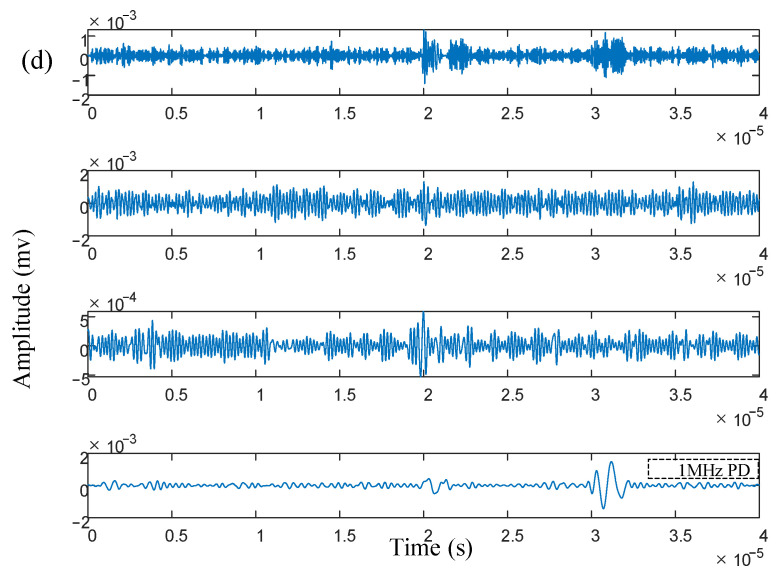
Four methods simulate signal decomposition results: (**a**) EWT, (**b**) AEFD, (**c**) VMD, (**d**) CEEMDAN.

The segmentation performance of IPSK was comparatively evaluated against four conventional methods (EWT, AEFD, VMD, and CEEMDAN), with their decomposition results presented in Figure 9a–d. For EWT, the number of modes is set to 4, and the local maxima segmentation mode is used. For AEFD, the initial boundary set is configured according to the spectrum of the original signal as [80, 300, 720], and the adaptive *ε*-neighborhood segmentation mode is employed. For VMD, the number of modes is set to 4, and the penalty parameter α is set to 2000.

As shown in Figure 9a,b, while both EWT and AEFD methods successfully isolated the 1 MHz PD signal in their first components, these components remained significantly contaminated by white noise. Moreover, the 10 MHz PD signal exhibited mode-mixing effects, being dispersed across the second and third components while simultaneously suffering from varying levels of narrowband interference, which substantially compromised signal feature identification. Figure 9c demonstrates that VMD could relatively clearly extract the 10 MHz PD signal in its second component. However, the 1 MHz PD signal was completely obscured by narrowband interference in the first component, rendering it unrecognizable. The decomposition results of Figure 9d CEEMDAN show that the fourth component is a 1 MHz PD signal, but it still contains white noise interference and has some distortion. The modal mixing of the other components is serious, and the 10 MHz PD signal cannot be effectively identified. In summary, compared with the EWT, VMD, CEEMDAN, and AEFD methods, IPSK achieves a superior decomposition accuracy while more effectively separating multi-source complex PD signals into two independent PD components.

To quantitatively compare the accuracy of the PD signals extracted by each method, three evaluation metrics are provided: signal-to-noise ratio (SNR), root-mean-square error (RMSE), and normalized correlation coefficient (NCC) [16]. Their calculation formulas are as follows:

(13) $$SNR=10\mathrm{lg}\left(\frac{\sum_{i=1}^{n}x^{2}\left(i\right)}{\sum_{i=1}^{n}{\left(x\left(i\right)-x^{\lambda }\left(i\right)\right)}^{2}}\right)$$

(14) $$RMSE=\sqrt{\frac{1}{n}\sum_{i=1}^{n}{\left(x^{\lambda }\left(i\right)-x\left(i\right)\right)}^{2}}$$

(15) $$NCC=\frac{E\left(\left(x-\mu_{x}\right)\left(x^{\lambda }-\mu_{x^{\lambda }}\right)\right)}{\sigma_{x}\sigma_{x^{\lambda }}}$$

In these formulas, $x\left(i\right)$ represents the original signal, and $x^{\lambda }$ represents the denoised signal. $\mu_{x}$ and $\mu_{x^{\lambda }}$ are the expectations of the signals before and after denoising, respectively. $\sigma_{x}$ and $\sigma_{x^{\lambda }}$ are the standard deviations of the signals before and after denoising, respectively. A higher SNR indicates more effective information contained in the PD signal; an RMSE value closer to 0 means less distortion of the pulse after denoising; and an NCC value closer to 1 indicates higher similarity between the denoised signal and the original signal. The evaluation metrics for each method are shown in Table 2.

The comparative results in Table 2 demonstrate that the IPSK method exhibits significant advantages across all evaluation metrics. In terms of the signal-to-noise ratio, IPSK (8.941 dB) shows an approximate 80% improvement over the second-best-performing AEFD (4.973 dB), indicating a superior noise suppression capability. Regarding signal fidelity, its RMSE value (1.311 × 10^−4^) is 27.6% lower than the worst-performing VMD, reflecting a higher signal reconstruction accuracy. Meanwhile, IPSK achieves an NCC value of 0.961, approaching the ideal value of 1 and significantly exceeding CEEMDAN’s 0.711, with virtually no waveform distortion observed.

These outstanding performance characteristics primarily stem from the IPSK method’s innovative incorporation of improved power spectrum segmentation technology based on spectral kurtosis. By precisely identifying and separating narrowband interference and white noise components in multi-source composite PD signals, it effectively addresses the insufficient decomposition accuracy of traditional methods when processing complex PD signals.

## 4. Analysis of Measured Signal

**Figure 10:**
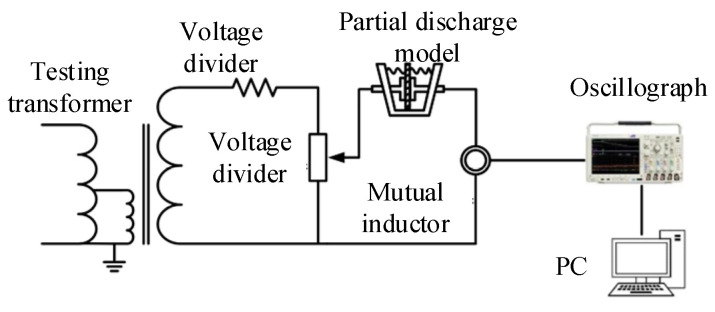
Experimental platform of partial discharge measurement system.

To validate the effectiveness of the proposed method for practical signal analysis, we established a PD detection experimental platform, as illustrated in Figure 10 [17] when adjusting the voltage on the test transformer through this platform. When the current flows through the discharge model, partial discharge will occur. To better simulate the complex electromagnetic environment encountered in actual working conditions, the experiment specifically introduced two typical interference models: corona discharge and surface discharge, in order to obtain multi-source composite PD signals that are representative of engineering applications.

**Figure 11:**
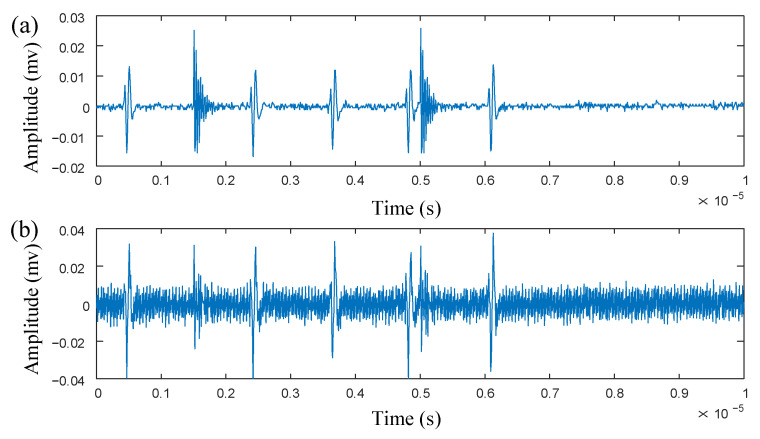
(**a**) Time-domain waveform of multi-source PD signal. (**b**) Time-domain waveform of noisy multi-source PD signal.

Due to significant discrepancies between laboratory conditions and actual operational environments, it is difficult to achieve the noise standards of a transformer during actual operation. Therefore, a periodic sinusoidal narrowband interference signal is added to the experimental signal, which has an amplitude of 9 mV and a frequency of 40 MHz. At the same time, Gaussian white noise with a signal-to-noise ratio of −2 dB is added. The final collected noisy multi-source PD signal is shown in Figure 11b.

**Figure 12:**
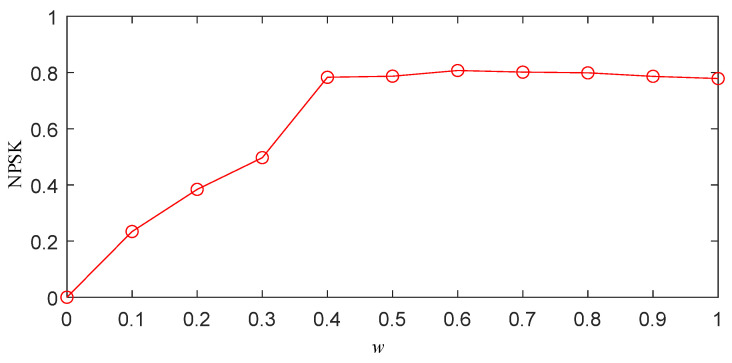
The normalized power spectral kurtosis of a noisy PD measured signal.

To obtain the frequency band division results of the IPSK method for the actual measured signals, it is necessary to determine the value of the parameter *w*. The specific procedure is the same as described in Section 2.3. The resulting NPSK is shown in Figure 12. From the figure, it can be observed that when *w* = 0.4, the kurtosis reaches its maximum value. Further increasing *w* beyond this point results in almost no change in the NPSK value. Therefore, the parameter *w* is set to 0.4.

**Figure 13:**
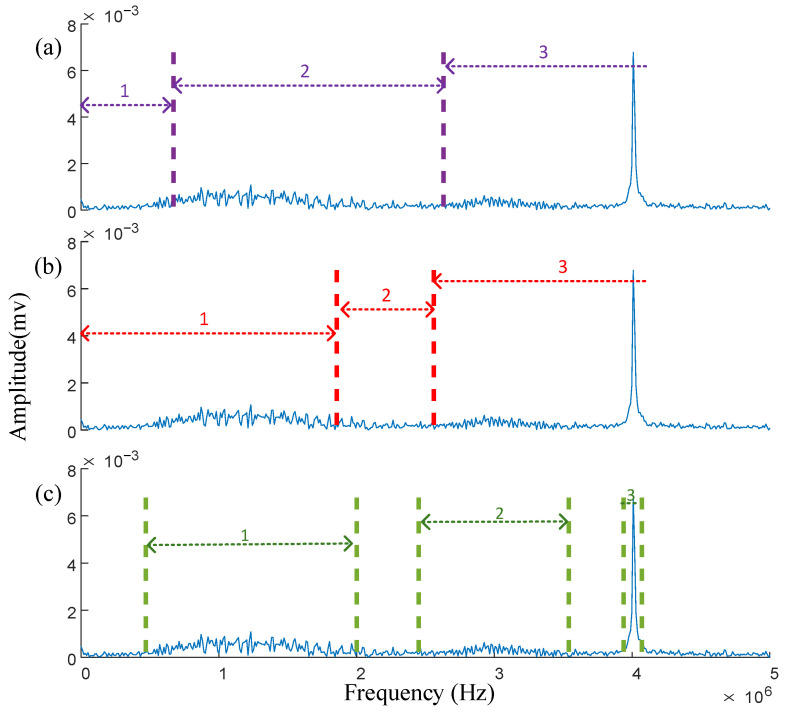
Comparison of spectrum segmentation results of three methods: (**a**) EWT, (**b**) AEFD, (**c**) IPSK.

By substituting the obtained *w* value into the threshold β, the power spectrum segmentation boundary of the actual measured signal can be obtained, and the EWT, AEFD, and IPSK are compared in terms of spectrum segmentation, as shown in Figure 13.

Figure 13a reveals that EWT divides the spectrum into three segments. However, the first segment contains only white noise without any valid signal components, representing an invalid partition. While the second segment successfully extracts the first PD signal, and the third segment mixes the second PD signal with narrowband interference. This segmentation approach induces significant mode mixing during subsequent time-domain analysis, severely compromising the accuracy of signal feature extraction. As shown in Figure 13b, AEFD similarly partitions the spectrum into three segments. The first segment correctly extracts the first PD signal, but the second segment contains minimal PD information with predominant noise, constituting another invalid partition. Like EWT, AEFD’s third segment combines the second PD signal with narrowband interference. In contrast, Figure 13c demonstrates that IPSK achieves non-contiguous spectral segmentation with well-defined, limited bandwidth intervals containing substantially less noise. This approach effectively eliminates most noise interference while successfully separating both PD signals, demonstrating clear advantages over conventional segmentation methods.

**Figure 14:**
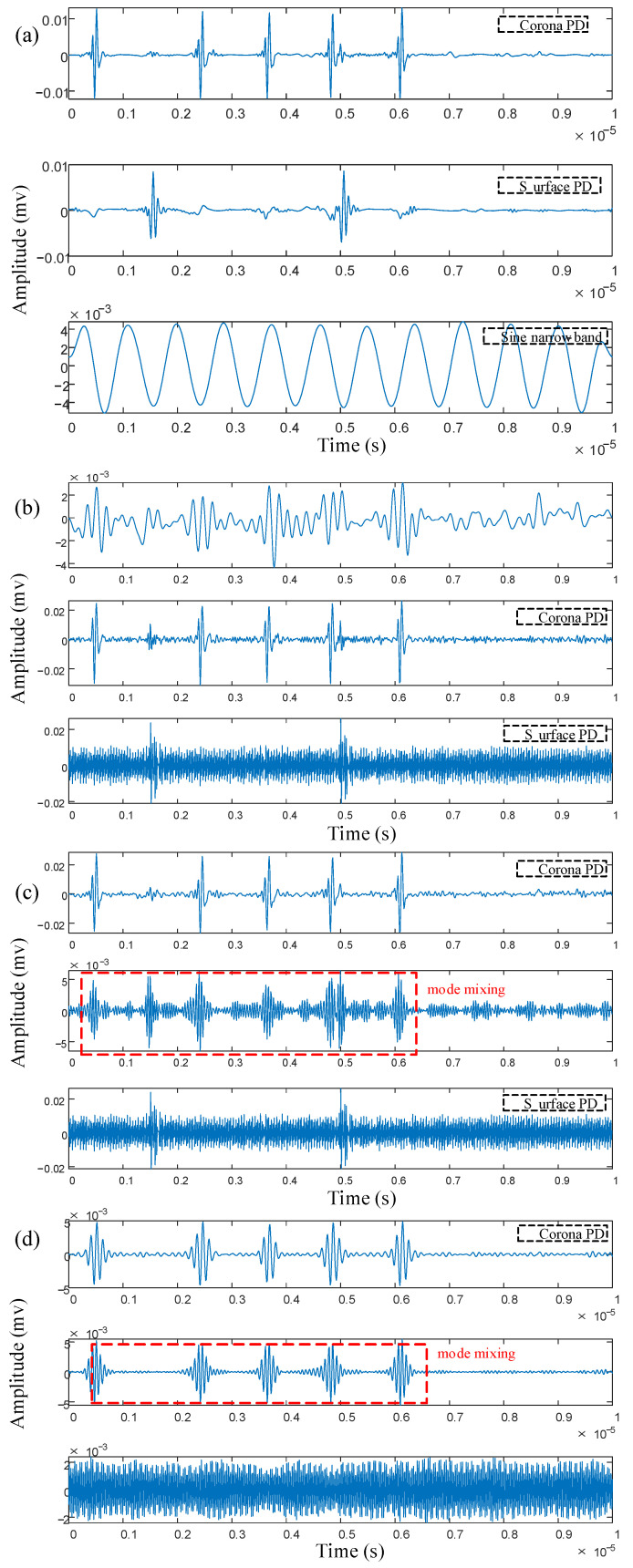

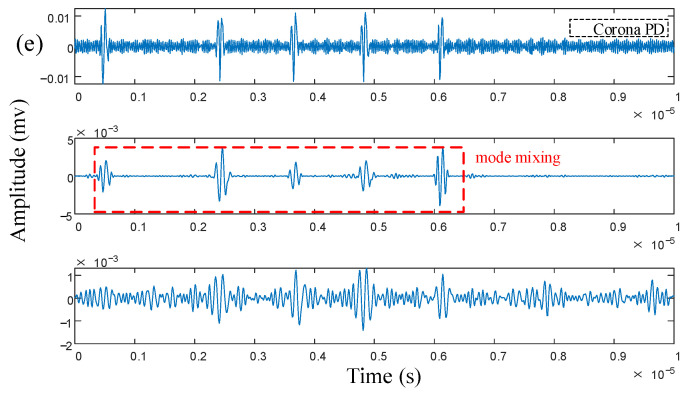
Decomposition results of measured signals by five methods: (**a**) IPSK, (**b**) EWT, (**c**) AEFD, (**d**) VMD, (**e**) CEEMDAN.

The segmentation performance of IPSK was compared with the EWT, AEFD, VMD, and CEEMDAN methods, with their respective reconstructed time-domain waveforms for each frequency band presented in Figure 14a–e. For EWT, the number of modes is set to 3, and the local maxima segmentation mode is used. For AEFD, the initial boundary set is configured according to the spectrum of the actual measured signal as [100, 270], and the adaptive ε-neighborhood segmentation mode is employed. For VMD, the number of modes is set to 3, and the penalty parameter *α* is set to 2000.

As shown in Figure 14a, the three components reconstructed by the IPSK method correspond to the corona PD signal, the surface PD signal, and the periodic sinusoidal narrowband interference signal. It can be observed from the figure that the boundaries of each component are clear, the features are distinct, and there is no modal mixing. This indicates that the method accurately decomposes the complex PD signals into two independent PD signals, achieving good results. From Figure 14b EWT, it can be seen that the first component decomposed by EWT is an invalid component, containing only some modal mixing and white noise. The second component is the corona PD signal, but it is heavily contaminated by white noise. The surface PD signal is located in the third component, but it is completely submerged in narrowband interference. The decomposition results of AEFD in Figure 14c are similar to those of EWT, both only identifying the corona PD signal and containing invalid components. In Figure 14d VMD, it can be observed that the corona PD signals are dispersed into the first and second components, where there is narrowband interference and waveform distortion, and the surface PD signals cannot be identified, resulting in the loss of effective information. Regarding Figure 14e CEEMDAN, the decomposition results indicate that the corona PD signals exhibit modal mixing in the first and second components, and the first component is severely affected by narrowband interference. Furthermore, the surface PD signals are not recognized in the CEEMDAN decomposition results. Thus, through the aforementioned experiments and analysis, the superiority of the IPSK method in processing actual measured signals is further confirmed.

## 5. Conclusions

This paper proposes an improved power spectrum kurtosis (IPSK) method based on spectral kurtosis and applies it to both simulated and actual measured signals. The conclusions drawn are as follows.

The IPSK method demonstrates a superior time-frequency resolution and decomposition accuracy compared to conventional time-frequency analysis techniques, enabling more effective suppression of both narrowband interference and white noise. In terms of critical performance metrics, the partial discharge signals extracted using IPSK exhibit remarkable advantages: achieving nearly 80% improvement in signal-to-noise ratio (SNR), 27.6% reduction in root-mean-square error (RMSE), and an outstanding normalized cross-correlation coefficient (NCC) of 0.961—approaching the ideal value while maintaining an exceptional waveform fidelity.

Through its innovative adaptive power spectrum segmentation technology, this method effectively addresses inherent limitations of traditional approaches including parameter sensitivity, mode mixing, and computational efficiency, thereby providing a more reliable solution for partial discharge detection in complex noisy environments.

## Figures and Tables

**Table 1:** Parameters of PD signal.

PD	$A_{i}$/mV	$\tau_{i1}$/μm	$\tau_{i2}$/μm	$f_{ci}$/MHz
$f_{1}\left(t\right)$	10	2	3	10
$f_{2}\left(t\right)$	3	2	3	1

**Table 2:** The evaluation index of *x*(t) by each method.

Methods	SNR/dB	RMSE	NCC
IPSK	8.941	$1.311\times 10^{-4}$	0.961
EWT	4.665	$1.630\times 10^{-4}$	0.856
AEFD	4.973	$1.592\times 10^{-4}$	0.879
VMD	3.692	$1.811\times 10^{-4}$	0.794
CEEMDAN	4.455	$1.602\times 10^{-4}$	0.711

## Data Availability

The original contributions presented in this study are included in the article. Further inquiries can be directed to the corresponding author.

## Abbreviations

The following abbreviations are used in this manuscript:

IPSK	Improved power spectral segmentation based on spectral kurtosis
PD	Partial discharge
EWT	Empirical wavelet transform
VMD	Variational mode decomposition
AEFD	Adaptive empirical Fourier decomposition
CEEMDAN	Complete ensemble empirical mode decomposition with adaptive noise
PSK	Power spectral kurtosis
NPSK	Normalized power spectral kurtosis
